# A Systematic Review and Meta-Analysis of Hospitalised Current Smokers and COVID-19

**DOI:** 10.3390/ijerph17207394

**Published:** 2020-10-11

**Authors:** Jesus González-Rubio, Carmen Navarro-López, Elena López-Nájera, Ana López-Nájera, Lydia Jiménez-Díaz, Juan D. Navarro-López, Alberto Nájera

**Affiliations:** 1 School of Medicine, CRIB, University of Castilla-La Mancha, 02008 Albacete, Spain; Jesus.Gonzalez@uclm.es; 2 Hospital General La Mancha Centro, Servicio de Salud de Castilla-La Mancha, Alcazar de San Juan, 13600 Ciudad Real, Spain; mdelnl@sescam.jccm.es; 3 Gerencia de Atención Primaria, Salud de Castilla y Leon, 05003 Avila, Spain; malopezna@saludcastillayleon.es; 4 Gerencia de Emergencias Sanitarias, 47407 Salud de Castilla y Leon, Spain; amlopezn@saludcastillayleon.es; 5 Centre for Biomedical Research, School of Medicine, University of Castilla-La Mancha, 13071 Ciudad Real, Spain

**Keywords:** COVID-19, SARS-CoV-2, cholinergic anti-inflammatory pathway, nicotine, cytokine release syndrome (CRS), current smokers

## Abstract

SARS-CoV-2 is a new coronavirus that has caused a worldwide pandemic. It produces severe acute respiratory disease (COVID-19), which is fatal in many cases, characterised by the cytokine release syndrome (CRS). According to the World Health Organization, those who smoke are likely to be more vulnerable to infection. Here, in order to clarify the epidemiologic relationship between smoking and COVID-19, we present a systematic literature review until 28th April 2020 and a meta-analysis. We included 18 recent COVID-19 clinical and epidemiological studies based on smoking patient status from 720 initial studies in China, the USA, and Italy. The percentage of hospitalised current smokers was 7.7% (95% CI: 6.9–8.4) in China, 2.3% (95% CI: 1.7–2.9) in the USA and 7.6% (95% CI: 4.2–11.0) in Italy. These percentages were compared to the smoking prevalence of each country and statistically significant differences were found in them all (*p* < 0.0001). By means of the meta-analysis, we offer epidemiological evidence showing that smokers were statistically less likely to be hospitalised (OR = 0.18, 95% CI: 0.14–0.23, *p* < 0.01). In conclusion, the analysis of data from 18 studies shows a much lower percentage of hospitalised current smokers than expected. As more studies become available, this trend should be checked to obtain conclusive results and to explore, where appropriate, the underlying mechanism of the severe progression and adverse outcomes of COVID-19.

## 1. Introduction

Severe acute respiratory syndrome coronavirus 2 (SARS-CoV-2), the new coronavirus that first broke out in Wuhan (Hubei Province, China) in December 2019, has quickly spread and become a global pandemic [1,2]. SARS-CoV-2 is the third coronavirus outbreak of this century, following severe acute respiratory syndrome coronavirus (SARS-CoV) and Middle East respiratory syndrome coronavirus (MERS-CoV) [3]. Coronavirus disease 2019 (COVID-19) causes clinical manifestations that range from mild respiratory symptoms to severe pneumonia, can be fatal in many cases, and is aggravated by cytokine release syndrome (CRS) or cytokine storm [4].

It has been well established that smokers are at a significantly high risk of chronic respiratory disease and acute respiratory infections, and current smokers are at more risk of developing influenza than non-smokers [5]. Smoking is also closely associated with MERS-CoV [6], but there is no clear evidence for this association with SARS-CoV-2 [7].

In today’s pandemic caused by coronavirus 2019 (COVID-19), some clinical characteristics have been described, but not without controversy about the effects of tobacco [8,9,10,11,12]. All this suggests that a smoking habit background is a poor prognosis factor in infected patients [10], or smokers could be more prone to contagion [13,14]. As evidence is lacking, the effect that tobacco has on contagions, the number of hospital admissions and the seriousness of smoking patients is unclear [14].

It is worth remembering that smoking kills around eight million people worldwide every year [15], irrespectively of any interaction with COVID-19, which is why smoking cessation is an urgent priority. Nonetheless, clinical data published until the time of the COVID-19 outbreak in China, as well as the first date made public in the USA [16,17] and Italy [18], indicate that the number of smokers hospitalised for COVID-19 was perceptibly lower than expected if we bear in mind the prevalence of smoking in these countries, and even despite the possible biases in reports [16,19,20].

In China, the mean proportion of smokers is 26.1%. Among males, 54.0% are current smokers, and only 2.6% among women [21]. In the USA, the proportion of smokers is 15.6% in males and 12.0% in females, with a combined proportion of 13.7% [22]. The proportion of smokers in Italy is 19%, with 23.3% in males and 15.0% in females [23]. So, a similar or higher percentage of current smokers hospitalised with SARS-CoV-2 is expected to appear, with males predominating.

As this virus has recently appeared, just a limited number of studies have evaluated the possible risk factors including the effect of tobacco. Most of them have been systematic reviews and meta-analyses focusing on the association between smoking, disease progression and severity of the outcomes for COVID-19 patients (largely showing a positive relation between these factors) [8,12,14,24,25,26,27,28,29,30,31,32,33,34,35]. However, to the best of our knowledge, just a few works have focused on studying the low prevalence of current smokers within hospitalised COVID-19 patients—mainly found in clinical data from China outbreak reports or early USA data—and more importantly, proposing potential pathophysiological explanations for these findings [36,37,38,39,40,41,42,43]. In this sense, nicotine or nicotinic receptor agonists were early proposed as plausible anti-inflammatory mediators acting on the immune system to counteract the “cytokine storm” found in severe SARS-CoV-2 infected patients [43]. In a current pandemic scenario, with no effective treatments available, any potential clue, that could open new therapeutic approaches, should be examined rigorously. Given the existing gaps in evidence, we carried out both a systematic review and a meta-analysis of studies about COVID-19, which extends existing information about a smoking habit (current smokers) to patients hospitalised in China, USA, and Italy, to evaluate the relation between smoking and hospitalization by COVID-19. Possible confounding factors for data interpretation are extensively discussed and the role of nicotine and the cholinergic anti-inflammatory pathway is deeply analysed.

## 2. Methods

### 2.1. Literature Search Strategy

**Figure 1:**
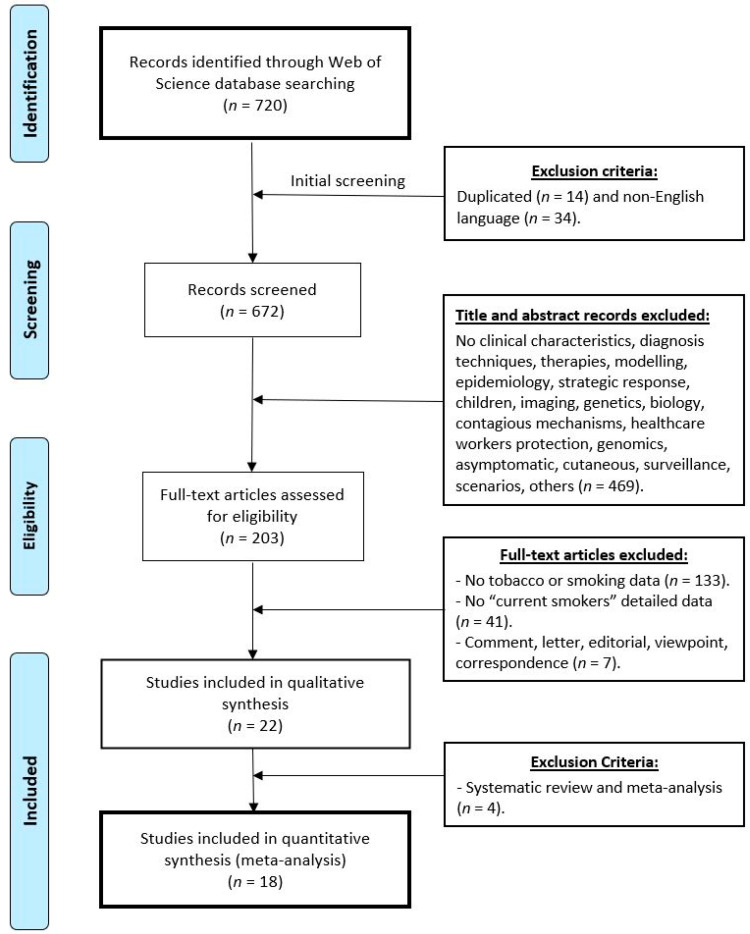
Flow chart diagram visualising the database searches, number of publications identified, screened, and final full texts included in the present systematic review and meta-analysis. Exclusion criteria are indicated.

The systematic review was carried out according to the Preferred Reporting Items for Systematic Review and Meta-Analysis (PRISMA) and Meta-analyses Of Observational Studies in Epidemiology (MOOSE) guidelines [44,45]. A flow chart is provided in Figure 1.

A systematic search was made of the ISI Web of Science (http://www.webofknowledge.com) for the relevant works published until 28 April 2020 (Figure 1; Identification phase) in any scientific field. Preprint databases were not used for the systematic search to assure that only peer-reviewed high-quality data were included in the subsequent meta-analysis. The following search terms were used: (‘COVID 19′ OR ‘NCOV 19′ OR ‘sars cov-2′ OR ‘sars cov 2′ OR ‘novel coronavirus’) AND (‘smoking’ OR ‘tobacco’ OR ‘smoker*’ OR ‘risk factor’ OR ‘clinical features’ OR ‘clinical characteristics’). The use of these terms assured the inclusion of any study related to coronavirus SARS-CoV-2 and hospitalised current smokers.

### 2.2. Inclusion and Exclusion Criteria

As described in Section 2.1, preprints were not included in the systematic review and meta-analysis, as only papers published in peer-reviewed journals were searched.

Figure 1 shows a flowchart diagram of the database searches and the exclusion/inclusion strategy followed. In a first phase (Figure 1; Screening phase), any duplicated works and those not written in English were excluded (Figure 1, Initial screening). Then, the studies that did not provide clinical characteristics were removed, or those describing diagnosis techniques, therapies, modelling, strategic response, imaging, genetics, biology, transmission mechanisms, healthcare workers protection, surveillance, scenarios, animal, genomics, those about asymptomatic patients, skin lesions and lesions specific of other organs, data on children or breastfed infants, among others. In the next phase (Figure 1; Eligibility phase), the works that did not provide details about smokers were removed, especially those with no data on “current smokers”. Finally, certain types of articles were excluded from the meta-analysis, e.g., comments, letters, editorial, viewpoint, correspondence, etc. (meta-analysis; Figure 1).

### 2.3. Data Extraction

Records were checked for duplicates using Zotero 5.0.85 (http://www.zotero.org). Two independent reviewers (AN and JGR) screened the literature search and assessed each study to be included by reading titles, abstracts and full texts. Any disagreement was solved in conference with the support of a third author (JN). Relevant data were acquired from each eligible study by means of a structured extraction sheet, which was prepared and approved by all the reviewers by reaching a consensus after screening the eligible studies.

### 2.4. Statistical Analysis

Data analyses were performed using meta packages in the R Software (R Software version 3.6.3; R Foundation, Viena, Austria; meta, dmetar and metafor). A random-effects meta-analysis was used to calculate the pooled estimated prevalence with 95% confidence intervals (95% CI). Achi-square test or Fisher’s exact test was carried out to compare the differences between the observed and expected current smokers for all the studies individually and by combining all the data.

In the present work, two meta-analyses were performed, one for data the extracted from studies in China and another for all the studies of the systematic review (i.e., also including studies from the USA and Italy). In both, the odds ratio (OR) represent the association between current smoking among hospitalised patients with COVID-19. Then, an OR = 1 indicates no association between variables. However, values < 1 indicate a negative association between the variables while values > 1 indicate a positive association. The further the odd ratio is from 1, the stronger the relationship between the studied variables.

*Heterogeneity*. Heterogeneity in a meta-analysis refers to the variation in the study outcomes between studies [46]. In the present work, the heterogeneity between studies was assessed by the Cochrane chi-squared test (χ^2^), Tau-squared (τ^2^), and I-squared (I^2^; Inconsistency). Depending on the I^2^ value, a fixed-effects (less than 50%) or a random-effects (more than 50%) model was used.

Several options are available if heterogeneity is identified between a group of studies [46], some of which have been considered in our meta-analyses: to verify if data are correct; to perform a meta-analysis of random effects (depending on the I^2^ value, a fixed-effects -less than 50%- or a random-effects -more than 50%- model was used); and to explore heterogeneity, and to exclude studies).

Another tool used to graphically study heterogeneity is *L’Abbé* plot [47], which represents the response rates to treatment versus the response rates in the control group and their position with respect to the diagonal. Studies are usually plotted with an area proportional to its accuracy, and its dispersion indicates heterogeneity. Therefore, *L’Abbé* graph allows us to check two important aspects of the meta-analyses performed in the present work: the general tendency of our meta-analysis and heterogeneity.

*Outliers.* A common method to detect outliers is to define a study as an outlier if its confidence interval does not overlap with the confidence interval for the pooled effect. This means that we defined a study as an outlier when its effect size estimate is so extreme that we have high certainty that the study cannot be part of the ‘population’ of the effect sizes that we actually pooled in our meta-analysis (that is, the individual study differs significantly from the overall effect). For the analysis of outliers, following the above premises, the R Software version 3.3.6, *find.outliers* function was used.

## 3. Results

### 3.1. Literature Retrieval

The literature search gave 720 articles (Figure 1, identification phase). Removing the duplicate documents (*n* = 14) and those not written in English (*n* = 34) left 672 items to be screened (Figure 1, screening phase). Then, the selection was performed by reading the titles and abstracts (469 were excluded). In total, 203 full text articles remained potentially eligible. Finally, publications were selected by applying the final selection criteria (detailed current smoker data and hospitalised patients). Of the remaining 203 works, 133 did not include smoking data and 41 did not include data about smoking habit or a smoking background. Comments, letters, viewpoint and editorials were also excluded (*n* = 7) and 22 works that detailed the data of the proportion of smokers by specifying current smokers and hospitalised patients remained eligible.

Finally, systematic reviews and meta-analyses articles were not included. This procedure gave 18 experimental documents: 15 papers with data on the China outbreak [48,49,50,51,52,53,54,55,56,57,58,59,60,61,62] (Table 1), one official report with preliminary data on the USA outbreak [16], one in New York city [17] and one for the Italian outbreak [18]. More details are provided in Table 1 and Table 2 to facilitate the interpretation of the analysed data as well as in the flow chart (Figure 1) to make this search repeatable in the future.

### 3.2. China

Fifteen studies of the total 18 selected in our systematic review search reported data from the China outbreak and were included in a specific meta-analysis (meta-analysis of studies from China) as described below (Section 3.3). As previously mentioned, all the studies included in the analysis contained detailed data about hospitalised current smokers. All the patients had been diagnosed with COVID-19 by PCR tests. Most studies were conducted in the Hubei province [48,50,52,54,57,58,60,61,62], three in the Zhejiang province [51,53,59], one in the Anhui province [56] and another in the Chongqing province [55]. One study collected data from 30 provinces [49] and from 522 hospitals. In general, most of the studies collected data from patients in only one hospital. Almost all the works included in the meta-analysis were retrospective, one was prospective [61] and one was ambispective [52]. Their collected data were taken between the 11 December 2019 and 12 February 2020. Data were generally taken from electronic medical records, except for one work, which collected them directly by personally communicating with patients [56]. The studies homogeneously reported clinical and epidemiological data, and included patients, for example, in the order in which they arrived at hospital. However, one of the studies included 17 patients who had been discharged from hospital [50] and included the highest percentage of current smokers (12.6%). Three other studies recruited patients according to some selection criterion, or because they presented abnormal imaging findings [59], had previously visited the Huanan seafood market [61] or were older patients [53].

Table 1 presents the data that correspond to the 15 included studies. They all provide details of the total proportion of males and females, and the number of current smokers. The expected smokers values were calculated with these details, the proportion of males and females in each study and the smoking prevalence in China [21]. The 95% confidence interval (95% CI) of the percentage of smokers estimated with the observed values was also included. In all cases, statistically significant differences (*p* < 0.001) appeared between the observed and expected values, except for the study by Han et al. 2020, whose sample included only 17 patients (*p* = 0.9999). The combined values were obtained by adding all the patients in each study to consider a total sample of 5023 patients, of whom 386 were current smokers. The prevalence percentage of current smokers was 7.7% (95% CI: 7.0–8.5%). Once again, the observed difference was very significant (*p* < 0.0001) compared to the expected values. This value was much lower than the expected one when considering the prevalence in China (54% in males, 2.6% in females, and a combined 26.1%).

**Figure 2:**
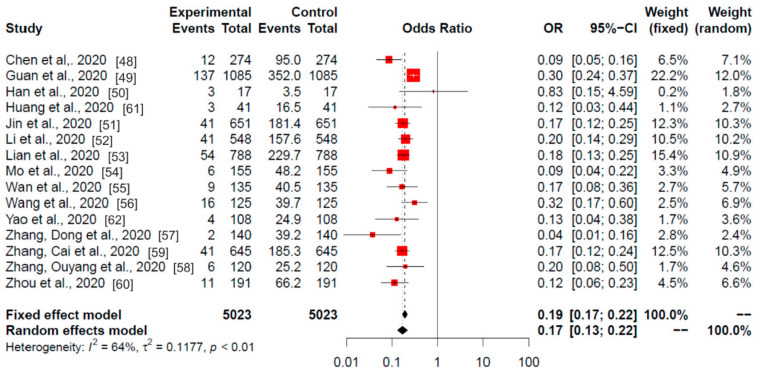
Meta-analysis of the Chinese studies. Odds ratios of the current smokers (experimental) among the hospitalised (control) patients with COVID-19 are shown. Data are from 15 published studies from the China outbreak. Red squares area is proportional to the size of the sample data. Black crosses and horizontal lines represent OR and 95% CI, respectively.

*Meta-Analysis of Studies from China.*Figure 2 offers meta-analysis results from the studies in China. 

The obtained heterogeneity (I^2^) was 64%, so the selected model was the random model (*p* < 0.01), which gave an odds ratio (OR) value of 0.17 and a 95% CI of 0.13–0.22. The OR results of the meta-analysis revealed statistically significant differences in 14 of the 15 studies. Only the study by Han et al. (2020) (study # 3; correspondence between numbers and studies can be found in Table 1, second column) did not show differences. These data suggest a strong negative association between the current smokers among hospitalised patients with COVID-19.

### 3.3. USA and Italy

Only three studies not conducted in China were included in our systematic review: two from the USA with official data from Centers for Disease Control and Prevention (CDC) and New York city [16,17], and one from Italy [18]. As the numbers are small, they are all presented in this section (Table 2). In all, the two US studies included 2412 hospitalised patients, of whom 55 were current smokers (1.7% and 5.1%, respectively), although no gender proportions were provided in the CDC study. The Italian study recruited 236 patients, of whom 18 were current smokers (7.6%). All the patients’ COVID-19 diagnosis had been confirmed by laboratory tests, in which case the US studies employed an official report [16] and a comment to the Editor [17] but provided detailed information about current smokers.

When comparing the observed and expected values according to the smoking prevalence in each country, the differences were very statistically significant in all cases (*p* < 0.0001). This result was also obtained when the expected proportion was analysed by considering the combination of the two US studies, suggesting a strong negative association between current smokers among hospitalised patients with COVID-19.

### 3.4. Global Meta-Analysis

**Figure 3:**
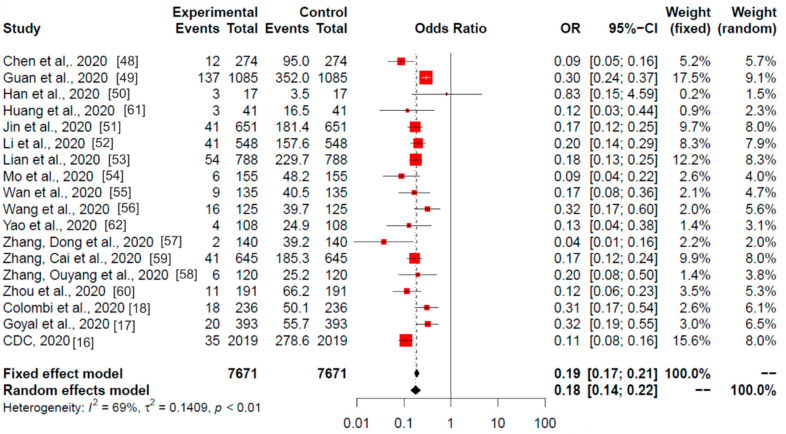
Global meta-analysis (China, Italy, and USA studies). Odds ratios of current smoking (experimental) among hospitalised (control) patients with COVID-19 are shown. The analysis included all (18) studies selected in the systematic review. Red squares area is proportional to the size of the sample data. Black crosses and horizontal lines represent OR and 95% CI, respectively.

Figure 3 provides the meta-analysis results of the 18 studies from China, USA and Italy included in the systematic review. The resulting heterogeneity was I^2^ = 69% (*p* < 0.01), so the random model that provided an OR of 0.18 and a 95% CI of 0.14–0.22 was selected.

The meta-analysis results (OR) revealed statistically significant differences in 17 of the 18 studies and in the combined total (*p* < 0.01). Similarly to the China meta-analysis (Figure 2), only one study did not show these differences, that by Han et al. (2020) [50].

### 3.5. Heterogeneity

**Figure 4:**
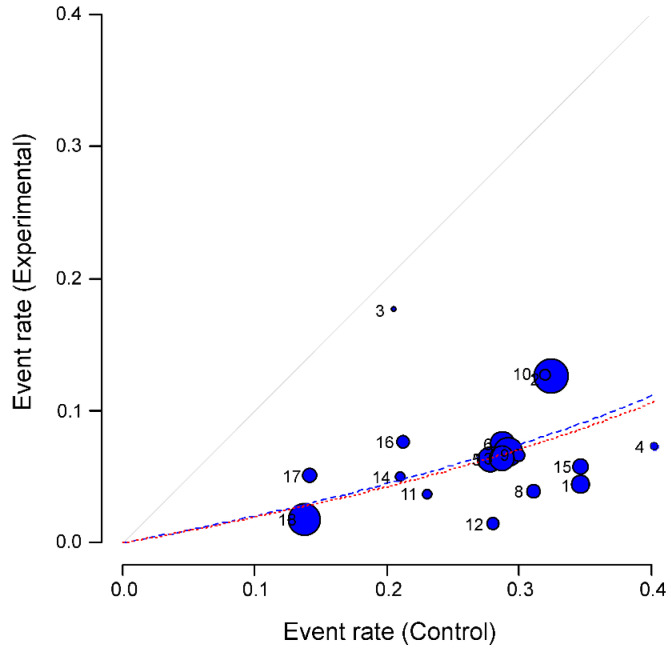
**Heterogeneity of the studies in the meta-analysis: L’Abbé graph** [47]. All 18 studies selected in the systematic review are plotted, numbered from 1 to 18 (correspondence between numbers and studies can be found above, Table 1 and Table 2, second column). The graph represents the response rates to the experimental event (current smoking) versus the response rates in the control group (hospitalization). Studies are plotted with an area proportional to its accuracy (blue circles), and its dispersion indicates heterogeneity. Dashed lines represent the pooled effect of the meta-analysis (red for fixed effect model and blue for random effect model).

As described in the methods section, *L’Abbé* graph [47] was used to further explore the heterogeneity of the 18 studies included in our global meta-analysis (15 studies from China, 2 from USA and 1 from Italy; Figure 4).

Results confirmed a protective effect of current smoking on the likelihood of hospitalisation for COVID-19 patients. *L’Abbé* plot showed that all the studies were located below the diagonal. Dashed lines symbolize the pooled effect of the meta-analysis (red and blue if fixed or random effect models were used, respectively).

The plot also allowed us to search for individual studies or groups of them that contributed to the heterogeneity of the effect already found. Specifically, the studies number (#) 2, #10 and #12 (see numbers assigned to each study in Table 1 and Table 2, second column) seem to follow less the tendency of the rest of the studies; as well as study number 3. However, only study #2 (corresponding to Guan et al., 2020 [49]) was found as an outlier. The same study was also the only outlier if only the 15 studies from China were included in the analysis.

When the outlier study #2 (i.e., the study from Guan et al., 2020 [49]) was eliminated from either the meta-analysis of the studies in China or from the global meta-analysis, the heterogeneity was lowered considerably (I^2^ = 36.5% for the set of Chinese works and I^2^ = 55.7% in the global meta-analysis). These two “new” meta-analyses provided 0.16 OR and 95%CI 0.14–0.18 values for the China meta-analysis (fixed model) and 0.17 OR and a 95% CI 0.14–0.21 for the global meta-analysis (random model).

## 4. Discussion

This work took data from 18 studies conducted in different parts of the world, but mainly China. They describe the number of current smokers hospitalised and with a confirmed COVID-19 diagnosis. All the studies included in the meta-analysis provide details of the patients’ smoking background, which allowed to determine the number of current smokers. This is very important because the other studies excluded from the analysis, despite having recruited lots of patients, did not provide information about smoking background [19].

In each case, these data were compared to the prevalence of smokers in each country by considering the proportion of males and females whenever possible. In every case except one, which had the fewest patients, very statistically significant differences were observed (*p* < 0.001) and would indicate that something is at play with regard to COVID-19 incidence in smokers.

To assure that only peer-reviewed data were analysed and therefore any bias due to poor-quality data was avoided in our meta-analyses, no pre-prints databases (vg. arXiv; bioarViv, etc.) were searched in the present work. However, we believe that, for future revisions, the inclusion of new studies and the extension of the search to other databases (vg. Pubmed), in which some current high-quality preprints would have developed into peer-reviewed works would be very interesting and beneficial.

Nevertheless, when a disease begins to spread in the population, the corresponding information is also transmitted between individuals, which in turn influences the pattern of the disease spread [63,64]. In this context, the responsible use and dissemination of some preprints may be of interest. Additionally, although there is a wide class of research that studies the dynamics of the dissemination of information, most of them are based on the classic spread of epidemics. Currently, the transmission of information requires a much lower cost and varies much faster than physical contagion, therefore the modelling of the dissemination of information should also help to understand the spread of epidemics [65] and the interpretation of the meta-analytic results of diseases.

### 4.1. Limitations and Biases

Both the systematic review and the presented meta-analyses have some limitations. Heterogeneity in the meta-analysis (i.e., variation in the study outcomes between the studies) was determined as I^2^ = 64% in the Chinese studies and as I^2^ = 69% when summing the US and Italian works. However, when heterogeneity was further explored through *L’Abbé* plotting, and the only outlier study was removed from both analyses, the I^2^ values decreased notably (I^2^ = 36.5% for the meta-analysis of Chinese works and I^2^ = 55.7% in the global meta-analysis) confirming the validity of our main results and conclusions.

It was not possible to perform a detailed study using the age groups of current smokers, although all patients were adults. As smoking habit prevalence changes with age, mean values were used. With males, this value could vary with age from 41.5% (males aged 70 years) and 60.3% (males between 40–49 years old) in China [20]. Conversely, these values for females were much lower, and varied between 1.2% (aged 18–29 years) and 5.8% (older than 70). The number of males and females was similar in practically all the studies. More male patients were included in all the studies, they smoked more heavily and were at higher risk of suffering the disease [66]. If tobacco, or some of its components, or smoking habit had some protective effect, more females would be expected to be hospitalised, but this was not the case. What we doubtlessly observed was that the difference between smokers hospitalised for COVID-19 and the expected values was very significant.

Other factors or artefacts could bias this study. For instance, as smokers know they are an at-risk population (as they are more likely to catch the disease from their habits: touching cigarettes and cigarette packets, exchanging tobacco, touching their face or placing cigarettes in their mouths, etc.; apart from the respiratory effects of tobacco itself), they could have been more aware of taking social distancing and hygiene measures. Nonetheless, as the temporal frame within which the studies were conducted was an early stage of today’s pandemic, and no differences were observed among them, this would not appear to be a plausible hypothesis.

Another possible bias may be related with data quality. We believe that smokers could have attempted to hide this characteristic given the alarm of these characteristics, and the threat of hospitals and ICUs being overcrowded. Nonetheless, most data were taken from electronic medical records, which meant that we had access to patients’ smoking background in many cases. Given the serious nature of the pandemic, in other cases we could presume that many smoking patients had stopped smoking before being hospitalised and were thus included in the groups of non-smokers or former smokers. So, it would be very interesting to specify the exact time when these data were collected, for example during a medical interview when admitted to hospital or from patients’ previous medical records. Moreover, the definition of “smoker” in such studies is not clear because heavy smokers are not distinguished from occasional smokers.

Epidemiologic studies could in some cases be inaccurate due to unrecognised bias. For example, while several case-control studies documented a “protective” effect of smoking on Alzheimer’s disease, subsequent cohort studies showed this was not the case and smoking may not be related to the onset of Alzheimer’s disease or possibly lead to a moderate increase in risk. Biased due to higher mortality in smoking AD patients, resulting in a lesser probability to catch them as cases in case-control studies was unveiled and could explain the inaccuracy [67]. In our work, data were included based on hospitalization records, therefore it was very unlikely that higher mortality in smoking COVID-19 patients would prevent them being selected in case-control studies biasing our meta-analysis. Current scientific evidence suggests that active smokers hospitalised for COVID-19 have a worse prognosis [14,30,37], and current smoking does not seem to be associated with an adverse outcome [24]. It must also be considered that current smokers cease to be so when entering the hospital, as far as nicotine is concerned. In our work, we only refer to the fact that there are less hospitalised current smokers than expected, which is why nicotine has been suggested to very likely have a protective effect against serious symptoms, calming the cytokine storm (see Section 4.2, [36,38,43]). This might be a cause of underrepresentation among hospitalised patients.

In any case, it is necessary to remember that tobacco causes 20,000 deaths a day all over the world [15] and, with COVID-19 patients, it generally comes with comorbidities, which means a worse prognosis [14].

Finally, in must be also noted that a potential threat to the validity of the meta-analytic results is the so-called *publication bias*, meaning that the publication of studies depends on the direction and statistical significance of the results. Studies with statistical significance are more likely to be published than those with non-significant results (which would be published less often) [68]. However, in the studies of the present meta-analyses, at the time of their publication, the fact that patients are smokers, ex-smokers or non-smokers is secondary information and therefore does not influence our results.

### 4.2. Physiological Substrate for Anti-Inflammatory Pulmonary Effect

SARS-CoV-2 causes varying degrees of illness. Fever and cough are the dominant symptoms, but severe disease also occurs. When COVID-19 patients’ aggravation takes place, lung hyperinflammation may appear due to a virus-activated “cytokine storm” or CRS [69]. Of the different cytokines that increase and reach such an exacerbated response [70], Interleukin-6 (IL-6) in serum is mainly expected to predict SARS-CoV-2 pneumonia severity as the suppression of pro-inflammatory IL-6 has been demonstrated to have a therapeutic effect on many inflammatory diseases, including viral infections [71]. In severe cases, SARS-CoV-2 has been shown to activate both innate and adaptive immune systems in alveolar tissue by inducing the release of many cytokines and subsequent cytokine release syndrome [72]. During this response, levels of pro-inflammatory cytokines (include TNFα, interleukin (IL)-1b, IL-6, and IL-8) rise [70], which is an important cause of death [73]. Therefore, it is believed that controlling such crucial inflammatory factors could be a successful approach to reducing mortality in severe patients.

The existence of a cholinergic anti-inflammatory pathway has been demonstrated, which modulates inflammatory responses during systemic inflammation [74]. The α7-nicotinic acetylcholine receptors (α7nAChR) are known to be expressed in macrophages and are essential for attenuating the inflammatory response by their activation during systemic inflammation [75]. The underlying mechanism conveys that α7nAChR activation in infiltrated inflammatory cells, including macrophages and neutrophils, induces not only the suppression of NF-kB activation [76], but also the secretion of pro-inflammatory cytokines and chemokines from inflammatory cells, including alveolar macrophages [77]. In lungs, this process involves a physiological feedback mechanism as it has been demonstrated that pulmonary injury signals produced by inflammation are transmitted by vagal sensory neurons to the central nervous system [78], where they are integrated and transformed into a vagal reflex [79]. This response activates the parasympathetic neurons innervated by the efferent vagus nerve, which results in a higher ACh concentration in the lungs [80]. Interestingly, it has been reported that nicotine, an α7nAChR agonist, exerts an anti-inflammatory effect of acute lung injury in a murine model [75]. In other inflammatory diseases, such as ulcerative colitis (UC), smoking or treatment with nicotine has been demonstrated to significantly reduce the risk of developing the disease [76]. Indeed, nicotine has been shown to reduce acute colonic inflammation severity with the concomitant inhibition of IL-6 mRNA expression [81,82,83]. So, nicotine, an exogenous α7nAchR agonist, has already been demonstrated to selectively downregulate the inflammatory response in a number of infection and inflammatory diseases and it has also been suggested that smoking could interact with susceptibility to SARS-CoV-2 infection through the renin–angiotensin system [84].

SARS-CoV-2 has been proposed to use the ACE2 receptor located at the surface of host cells to facilitate virus entry [85]. On the one hand, it has been suggested that smoking may upregulate ACE2 expression [13] and also that SARS-CoV-2 infections could be positive feed-back loops to increase ACE2 levels and facilitate virus dissemination [86]. On the other hand, evidence suggests that nicotine downregulates ACE2 expression [13,84,87]. In any case, the exact role of ACE2 as a mediator of disease severity remains to be determined. As ACE2 expression is necessary and sufficient for SARS-CoV-2 infections, it seems highly likely that an expansion of ACE2-expressing cells in the lungs facilitates viral shedding. However, it is possible that ACE2 expression also has some beneficial consequences. ACE2 has strong vasodilatory, anti-inflammatory, and antioxidant properties. Based on these properties, increased ACE2 levels have been proposed to be more beneficial than harmful, particularly in patients with lung injury. In this sense, children, and younger adults, who have milder COVID-19 symptoms, have higher ACE2 levels compared to older people [38]. Therefore, even if smoking upregulates ACE2, this does not necessarily imply an adverse prognosis [39,88]. For the above reasons, further research will be required to determine the precise impact of ACE2 levels on the clinical course of COVID-19 and its relationship to smoking and nicotine.

## 5. Conclusions

The number of hospitalised smokers was smaller than expected based on the smoking prevalence in the different countries. The meta-analysis results obtained in China, the US and Italy indicated that a smoking habit lowers the likelihood of being hospitalised by COVID-19.

Currently, the most promising trial run to treat severe COVID-19 patients is the one using Tocilizumab, a blocker of the IL-6 receptor for the treatment of cytokine storm [71]. However, very strict criteria for clinical use limits its availability, mainly due to its price and adverse effects. Another recent strategy has proposed the use of Baricitinib, which is predicted to reduce the ability of the virus to infect lung cells through the ACE2 receptor [89], although drugs with a similar action mechanism used in oncology bring serious side effects [89,90]. Nevertheless, to our knowledge, no clinical trials of nicotine in COVID-19 patients are currently being run. We suspect that nicotine could contribute to an amelioration of the cytokine storm and the severe related inflammatory response through the α7nAChR-mediated cholinergic anti-inflammatory pathway during a patient’s aggravation [43]. Hence, therapeutic strategies should probably consider the combination of antiviral and anti-inflammatory treatments [91] in order to reduce viral infectivity, viral replication, exacerbated inflammatory response, and to limit side effects.

## Figures and Tables

**Table 1:** Comparison of the hospitalised current smokers in the Chinese COVID-19 outbreak. Fifteen studies are described. The combined analysis is the result of adding the 15 individual studies. For each study, the number of male and female hospitalised patients, current smoker patients, 95%CI calculated with Wilson’s procedure, expected current smokers both pooled and by gender, and statistical significance (Sig.; *p*) are shown. Expected current smokers were estimated using 54% and 2.6% for males and females, respectively [21]. A column with a consecutive numbering of the studies (#) is also included.

Study	#	*n* (Male/Female)	Current Smokers	95%CI (Wilson)	Expected Current Smokers (Male/Female)	Sig.
Chen et al., 2020 [48]	1	274 (171, 103)	12 (4.4%)	(2.6–7.4)	95.0 (92.3, 2.7)	*p* < 0.0001
Guan et al., 2020 [49]	2	1085 (631, 454)	137 (12.6%)	(10.8–14.7)	352.5 (340.7, 11.8)	*p* < 0.0001
Han et al. 2020 [50]	3	17 (6, 11)	3 (17.6%)	(8.5–38.7)	3.5 (3.2, 0.3)	*p* = 0.9999
Huang et al., 2020 [61]	4	41 (30, 11)	3 (7.3%)	(3.6–18.3)	16.5 (16.2, 0.3)	*p* = 0.0006
Jin et al., 2020 [51]	5	651 (320, 331)	41 (6.3%)	(4.7–8.4)	181.4 (172.8, 8.6)	*p* < 0.0001
Li et al., 2020 [52]	6	548 (279, 269)	41 (7.5%)	(5.6–10.0)	157.7 (150.7, 7.0)	*p* < 0.0001
Lian et al., 2020 [53]	7	788 (407, 381)	54 (6.9%)	(5.3–8.8)	229.7 (219.8, 9.9)	*p* < 0.0001
Mo et al., 2020 [54]	8	155 (86, 69)	6 (3.9%)	(2.0-8.0)	48.2 (46.4, 1.8)	*p* < 0.0001
Wan et al., 2020 [55]	9	135 (72, 63)	9 (6.7%)	(3.8–12.0)	40.5 (38.9, 1.6)	*p* < 0.0001
Wang et al. 2020 [56]	10	125 (71, 54)	16 (12.8%)	(8.2–19.6)	39.7 (38.3, 1.4)	*p* = 0.0003
Yao et al., 2020 [62]	11	108 (43, 65)	4 (3.8%)	(1.8–8.7)	24.9 (23.2, 1.7)	*p* < 0.0001
Zhang, Dong et al., 2020 [57]	12	140 (69, 71)	2 (1.4%)	(0.8–4.6)	39.1 (37.3, 1.9)	*p* < 0.0001
Zhang, Cai et al., 2020 [59]	13	645 (328, 317)	41 (6.4%)	(4.7–8.5)	185.4 (177.2, 8.2)	*p* < 0.0001
Zhang, Ouyang et al., 2020 [58]	14	120 (43, 77)	6 (5.0%)	(2.6–10.2)	25.2 (23.2, 2.0)	*p* = 0.0004
Zhou et al., 2020 [60]	15	191 (119, 72)	11 (5.8%)	(3.4–9.9)	66.2 (64.3, 1.9)	*p* < 0.0001
Combined	-	5023 (2675, 2348)	386 (7.7%)	(7.0–8.5)	1505.6 (1444.5, 61.0)	*p* < 0.0001

**Table 2:** Comparison of the hospitalised current smokers in the COVID-19 outbreaks in the USA and Italy. A column with a consecutive number of the studies (#) is included. For each study, the number of male and female hospitalised patients, currently smoking patients, 95%CI calculated with Wilson’s procedure, expected current smokers to be both pooled and by gender (except for study #16), and statistical significance (Sig.; *p*) is shown. To calculate the expected current smokers’ values in the USA, 15.6% in males and 12.0% in females were taken, which gave a combined 13.7% [22]. In Italy, 23.3% in males and 15.0% in females were taken [23]. ^1^ Gender proportions are not specified.

Study	#	*n* (Male/Female)	Current Smokers	95%CI (Wilson)	Expected Current Smokers (Male/Female)	Sig.
CDC, 2020 [16]	16	2019 ^1^	35 (1.7%)	(1.3–2.4)	278.6 ^1^	*p* < 0.0001
Goyal et al., 2020 [17]	17	393 (238, 155)	20 (5.1%)	(3.4–7.7)	55.7 (37.1, 18.6)	*p* < 0.0001
USA, combined	-	2412	55 (2.3%)	(1.8, 3.0)	334.3	*p* < 0.0001
Colombi et al., 2020 [18]	18	236 (177, 59)	18 (7.6%)	(5.0–11.6)	50.1 (41.2, 8.9)	*p* < 0.0001
